# Comprehensive gene expression profiling identifies distinct and overlapping transcriptional profiles in non-specific interstitial pneumonia and idiopathic pulmonary fibrosis

**DOI:** 10.1186/s12931-018-0857-1

**Published:** 2018-08-15

**Authors:** Matthew J. Cecchini, Karishma Hosein, Christopher J. Howlett, Mariamma Joseph, Marco Mura

**Affiliations:** 1 Department of Pathology, Western University, London, Canada; 2 Division of Respirology, London Health Science Centre, Victoria Hospital, Western University, 800 Commissioners Road East Room E6-203, London, ON N6A 5W9 Canada; 3 Toronto Lung Transplant Program, University of Toronto, Toronto, Canada

**Keywords:** Idiopathic pulmonary fibrosis, Usual interstitial pneumonia, Non-specific interstitial pneumonia, Microarray

## Abstract

**Background:**

The clinical-radiographic distinction between idiopathic pulmonary fibrosis (IPF) and non-specific interstitial pneumonia (NSIP) is challenging. We sought to investigate the gene expression profiles of IPF and NSIP vs. normal controls.

**Methods:**

Gene expression from explanted lungs of patients with IPF (*n* = 22), NSIP (*n* = 10) and from normal controls (*n* = 11) was assessed. Microarray analysis included Significance Analysis of Microarray (SAM), Ingenuity Pathway, Gene-Set Enrichment and unsupervised hierarchical clustering analyses. Immunohistochemistry and serology of proteins of interest were conducted.

**Results:**

NSIP cases were significantly enriched for genes related to mechanisms of immune reaction, such as T-cell response and recruitment of leukocytes into the lung compartment. In IPF, in contrast, these involved senescence, epithelial-to-mesenchymal transition, myofibroblast differentiation and collagen deposition. Unlike the IPF group, NSIP cases exhibited a strikingly homogenous gene signature. Clustering analysis identified a subgroup of IPF patients with intermediate and ambiguous expression of SAM-selected genes, with the interesting upregulation of both NSIP-specific and senescence-related genes. Immunohistochemistry for p16, a senescence marker, on fibroblasts differentiated most IPF cases from NSIP. Serial serum levels of periostin, a senescence effector, predicted clinical progression in a cohort of patients with IPF.

**Conclusions:**

Comprehensive gene expression profiling in explanted lungs identifies distinct transcriptional profiles and differentially expressed genes in IPF and NSIP, supporting the notion of NSIP as a standalone condition. Potential gene and protein markers to discriminate IPF from NSIP were identified, with a prominent role of senescence in IPF. The finding of a subgroup of IPF patients with transcriptional features of both NSIP and senescence raises the hypothesis that “senescent” NSIP may represent a risk factor to develop superimposed IPF.

**Electronic supplementary material:**

The online version of this article (10.1186/s12931-018-0857-1) contains supplementary material, which is available to authorized users.

## Background

Idiopathic pulmonary fibrosis (IPF) and non-specific interstitial pneumonia (NSIP) are the most common forms of idiopathic interstitial pneumonia (IIP) [1]. Better prognosis and response to therapy are reported for NSIP compared to IPF, which is defined by a histologic pattern of usual interstitial pneumonia (UIP) [2]. However, both conditions represent a common indication for lung transplantation (LTx) [3]. While the fibrotic process in IPF is believed to be driven by alveolar injury leading to unresolving wound healing and pro-fibrotic signals [4], the pathogenesis of NSIP is not clear.

The clinical-radiographic distinction is challenging [5], but is particularly important given the differences in prognosis and treatment algorithms. Patients with NSIP will often have a good response to corticosteroids, while IPF can worsen on prednisone [6], and is currently treated with anti-fibrotic agents [7, 8]. This differential response to treatment further highlights the dissimilarities in the molecular basis that defines IPF and NSIP. Despite these differences, the frequent finding of mixed UIP-NSIP patterns on lung biopsies [9] supported the hypothesis that NSIP may represent an early form of IPF [10].

Differential diagnosis between IPF and idiopathic NSIP is based on high-resolution chest CT scan (HRCT) and pathology. Diagnostic radiographic criteria for NSIP are not clearly defined [11], and as many as 25% of patients with IPF present with HRCT features atypical for UIP [12]. Even with biopsies taken from multiple lobes, interobserver agreement in IIP is only moderate [13]. The finding of fibroblastic foci in cases of NSIP [14] adds more difficulty to the diagnostic process. Currently, multi-disciplinary discussion represents the gold standard approach in interstitial lung disease (ILD) [10], but its accuracy has never been validated. This highlights a clear need for more refined diagnostic tools.

Given the difference in outcomes and response to therapy, we hypothesized that IPF and NSIP exhibit distinct transcriptional profiles, and that specific gene markers may be identified for each condition. As Rosas and Kaminski pointed out, gene expression studies have been highly effective in reclassifying clinically relevant disease phenotypes with similar histologic presentation [15]. The authors also recommended a systems-level, rather than “cherry-picking” approach when analyzing microarray data [15]. In this study, we sought to investigate the gene expression profiles of IPF, NSIP and normal controls. Although we were interested in identifying individual genes and processes that could be candidates as disease markers, we opted for an integrated approach, founded on the analysis of biologically meaningful sets of genes, function and pathways. Using normal controls, we aimed at identifying genes that were specifically increased in each condition, and that could be used in the future in the differential diagnosis process.

## Methods

### Subjects

Specimens were obtained from the peripheral area of the lower lobe of each lung as soon as the first recipient lung was taken out, snap frozen in liquid nitrogen, and stored at − 80 °C. RNA was extracted and hybridized to the Human Gene 1.0 set array (Affymetrix) from explanted lungs (2001–2008) in 22 patients with clinical diagnosis of sporadic IPF, entirely typical UIP HRCT pattern [16] and definite histologic UIP pattern; 10 subjects with clinical diagnosis of idiopathic NSIP and definite histologic pattern of fibrotic NSIP; and 11 normal lung samples (age 52 ± 18 years, 4 females) obtained from the region of normal tissue flanking lung cancer resections in ILD-free patients. Histopathologic diagnoses were based on whole explanted lungs. IPF cases with atypical radiographic features for UIP, and patients with other types of ILD, connective tissue disease or concomitant emphysema were excluded. A separate set of patients who underwent surgical lung biopsies at London Health Science Centre (Western University) (2005–2015) were identified and representative blocks were used for immunohistochemistry (IHC). This set included 23 cases with definite IPF/UIP and 13 with definite NSIP.

The study was approved by the Human Tissue Committees and Research Ethics Boards of the University Health Network (protocol n.11–0932) and Western University (n.105214).

### Microarray analysis, immunohistochemistry and serum measurements

RNA was isolated, labeled, and hybridized to the human gene 1.0 set array according to the manufacturer’s protocols (Affymetrix). Data sets for microarray experiments are available at the Gene Expression Omnibus repository, accession n.GSE110147.

Partek software (St.Louis, MO) was used for the preliminary analysis. The q-value (false discovery ratio, used for multiple comparison correction) was used to identify differentially expressed genes and was computed with significance analysis of microarray (SAM). For pathways/networks discovery analysis, Ingenuity Pathway Analysis (IPA; Redwood City, CA) was used. For Gene-set Enrichment Analysis (GSEA), C5 GeneOntology was used as gene-set database [17, 18]. For hierarchic clustering, Cluster 3.0 and Treeview (Eisen’s Laboratory, Stanford University) were used.

All IHC slides were reviewed by 2 pathologists. IHC for periostin (POSTN), osteopontin (OPN), p53 and p16, selected ad hoc based on microarray results, was performed using standard techniques.

The serum levels of 6 proteins, a priori selected based on literature review (POSTN, OPN, surfactant protein-A [SP-A], matrix metallopeptidase-9 [MMP-9], Krebs von den Lungen-6 [KL-6], Chemokine-(C-C motif)ligand-18 [CCL-18]) were measured at baseline and at 4-month intervals for 12 months in 34 patients diagnosed with IPF [16] and followed at Western University. The endpoint of the longitudinal study was clinical progression, defined as either: > 10% absolute reduction in forced vital capacity % predicted; > 50 m decline in 6-min walk distance; hospitalization for respiratory causes; LTx assessment; or death.

Details on sample processing, RNA isolation, complementary DNA synthesis, pre-processing on probe-level data, IPA, GSEA, RT-PCR, IHC and serum measurements are provided in the Additional files 1, 2, 3, 4 and 5.

### Statistical analysis

The Kolmogorov-Smirnov test assessed variables’ distribution. For comparison of groups, either unpaired t test or the Mann-Whitney test, where indicated, was used. Cox proportional hazards regression analysis was used to identify serum biomarkers significantly predicting clinical progression. Receiver operating characteristic (ROC) analysis was used to determine the accuracy of serum biomarkers in predicting clinical progression (c-statistics). *P*-values < 0.05 were regarded as significant. Prism-4 software package (GraphPad, La Jolla, CA) was used.

## Results

There were no significant differences in pulmonary function tests, exercise capacity or pulmonary artery pressures. NSIP patients were significantly younger than IPF patients (Table 1).

**Table 1:** Demographic, clinical and functional characteristics of the patients included in the study

Variable	IPF (*n* = 22)	NSIP (*n* = 10)	*p* value
Male/Female (% males)	17/5 (77%)	2/8 (20%)	0.0051
Age (years)	62 ± 6	45 ± 11	< 0.0001
BMI (m/Kg^2^)	26 ± 5	24 ± 3	n.s.
mPAP (mmHg)^+^	29 ± 12	35 ± 21	n.s.
6MWD (m)	293 ± 103	292 ± 195	n.s.
FVC (% pred)	57 ± 19	49 ± 18	n.s.
TLC (% pred)	65 ± 14	65 ± 18	n.s.
DLCO (% pred)	37 ± 10	51 ± 18	0.0242
Treatment (% of total):			
Prednisone alone	7 (31.8%)	5 (50%)	n.s.
Prednisone + Azathioprine	8 (36.4%)	2 (20%)	n.s.
NAC alone	1 (4.5%)	0 (0%)	n.s.
No specific treatment	6 (27.3%)	3 (30%)	n.s.

^+^intraoperative mPAP during lung transplant

*IPF* idiopathic pulmonary fibrosis, *NSIP* non-specific interstitial pneumonia, *BMI* body mass index, *mPAP* mean pulmonary arterial pressurem, *6MWD* 6-min walk distance, *FVC* forced vital capacity, *TLC* total lung capacity, *DLCO* diffusing lung capacity for carbon monoxide, *n.s.* not significant

**Fig. 1:**
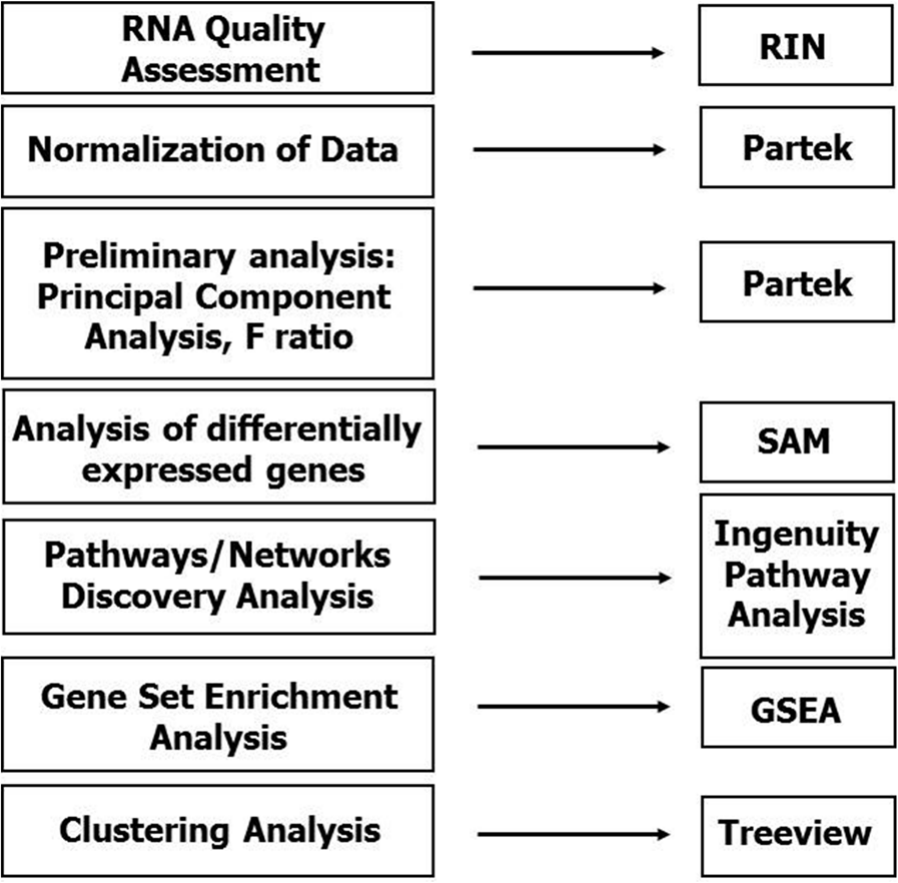
Outline of microarray analysis. SAM = Significance Analysis of Microarray. RIN = RNA integrity number

RNA integrity numbers were very similar in the 3 groups: 8.5 ± 0.4 in IPF, 8.8 ± 0.5 in NSIP and 8.6 ± 0.4 in normal controls. Sequential steps in gene expression profiling are shown in Fig. 1. There were no outliers among samples examined in terms of probe intensity (Additional file 1: Figure S1A). The F-ratio (signal-to-noise) ratio (average signal for all genes) was 1.77 (Additional file 1: Figure S1B). Principal Component Analysis (PCA, Additional file 2: Figure S2) showed a sizable, but not major degree of similarity across the genome between IPF and NSIP.

### Gene expression profile of IPF

With stringent selection criteria (fold change ≥1.50, q-value< 5%), SAM identified 146 differentially expressed genes, of which 60 upregulated in IPF and 86 upregulated in NSIP. The top 25 upregulated genes in each condition are shown in Tables 2 and 3.

**Table 2:** Top 25 up-regulated genes in IPF vs. NSIP (SAM analysis)

NCBI Gene Symbol	NCBI Gene name	d	Fold change vs. NSIP group	q value
IGFBP5	insulin like growth factor binding protein 5	4.70	1.78	< 0.0001
SLN	sarcolipin	4.69	2.57	< 0.0001
SYNPO2	synaptopodin 2	4.61	2.08	< 0.0001
MYH11	myosin, heavy chain 11, smooth muscle	4.38	1.99	< 0.0001
DES	desmin	4.33	2.05	< 0.0001
NLGN4Y	neuroligin 4, Y-linked	4.24	2.55	< 0.0001
FAM83D	family with sequence similarity 83, member D	4.23	1.65	< 0.0001
ACTG2	actin, gamma 2, smooth muscle, enteric	4.16	2.25	< 0.0001
TPM2	tropomyosin 2 (beta)	4.13	1.62	< 0.0001
CNN1	calponin 1, basic, smooth muscle	4.06	1.77	< 0.0001
PRUNE2	prune homolog 2 (Drosophila)	4.01	1.84	< 0.0001
EIF1AY	eukaryotic translation initiation factor 1A, Y-linked	4.00	7.99	< 0.0001
RPS4Y1	ribosomal protein S4, Y-linked 1	4.00	7.25	< 0.0001
AHNAK2	AHNAK nucleoprotein 2	3.93	1.76	< 0.0001
DDX3Y	DEAD (Asp-Glu-Ala-Asp) box helicase 3, Y-linked	3.91	11.4	< 0.0001
KDM5D	lysine (K)-specific demethylase 5D	3.89	5.23	< 0.0001
TXLNGY	taxilin gamma pseudogene, Y-linked	3.89	7.08	< 0.0001
ATP1A2	ATPase, Na+/K+ transporting, alpha 2 polypeptide	3.87	1.66	< 0.0001
ACTA-2	actin, alpha 2, smooth muscle, aorta	3.87	1.51	< 0.0001
TXLNGY	taxilin gamma pseudogene, Y-linked	3.86	5.71	< 0.0001
ZFY	zinc finger protein, Y-linked	3.79	4.13	< 0.0001
PDLIM3	PDZ and LIM domain 3	3.79	1.55	< 0.0001
TAGLN	transgelin	3.77	1.60	< 0.0001
UTY	ubiquitously transcribed tetratricopeptide repeat	3.72	7.80	< 0.0001
IGFBP6	insulin like growth factor binding protein 6	3.66	1.60	< 0.0001

**Table 3:** Top 25 up-regulated genes in NSIP vs. IPF (SAM analysis)

NCBI Gene Symbol	NCBI Gene name	d	Fold change vs. NSIP group	q value
LRP2	LDL receptor related protein 2	4.49	2.12	< 0.0001
IFI44L	interferon-induced protein 44-like	3.99	2.67	< 0.0001
SLC39A8	solute carrier family 39 (zinc transporter), member 8	3.97	2.67	0.0237
SCN1A	sodium channel, voltage gated, type I alpha subunit	3.96	1.97	0.0237
LNX2	ligand of numb-protein X 2	3.86	1.52	0.0237
WARS	tryptophanyl-tRNA synthetase	3.82	1.63	0.0237
SLC6A14	solute carrier family 6 (amino acid transporter), member 14	3.76	2.54	0.0237
FZD5	frizzled class receptor 5	3.76	1.61	0.0237
PCDH9	protocadherin 9	3.71	1.53	0.0237
F11	coagulation factor XI//4q35	3.68	2.50	0.0237
RSAD2	radical S-adenosyl methionine domain containing 2	3.63	2.26	0.0237
NECAB1	N-terminal EF-hand calcium binding protein 1	3.60	2.48	0.0237
MFSD2A	major facilitator superfamily domain containing 2A	3.57	1.97	0.0237
SDR16C5	short chain dehydrogenase/reductase family 16C, member 5	3.50	2.23	0.0237
MOP-1	MOP-1	3.48	1.60	0.0237
OAS-2	2–5-oligoadenylate synthetase 2	3.43	1.74	0.0237
PHACTR1	phosphatase and actin regulator 1	3.43	1.55	0.0237
PIGA	phosphatidylinositol glycan anchor biosynthesis class A	3.42	1.51	0.0237
IFIT3	interferon-induced protein with tetratricopeptide repeats 3	3.42	1.96	0.0237
C1orf162	chromosome 1 open reading frame 162	3.40	1.54	0.0237
ATP8A1	ATPase, aminophospholipid transporter (APLT), class I, type 8	3.40	1.52	0.0237
FMO5	flavin containing monooxygenase 5	2.44	2.44	0.0237
GBP4	guanylate binding protein 4	3.34	1.92	0.0237
ZNF385B	zinc finger protein 385B	3.32	2.36	0.0237
SLCO4C1	solute carrier organic anion transporter family, member 4C1	3.25	2.11	0.0237

d = standardized change in expression (relative difference); q value = false discovery ratio

**Fig. 2:**
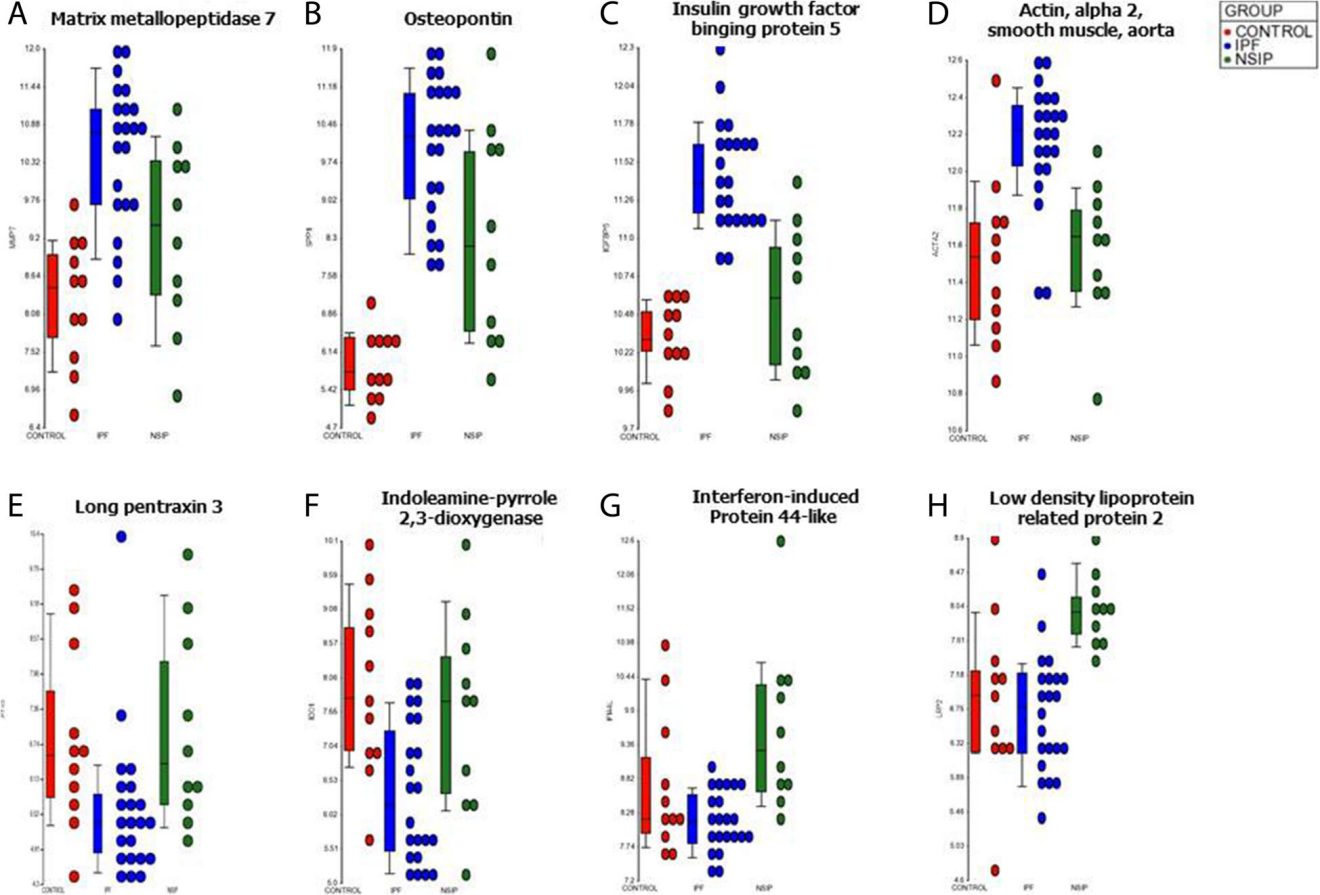
Gene expression levels determined by oligonucleotide microarray in the IPF (blue), NSIP (green) and normal control (red) groups. Examples of genes upregulated in both IPF and NSIP: **a**. Matrix Metalloproteinase 7 (MMP-7). **b** Osteopontin (also known as Secreted Phosphoprotein 1). Examples of genes specifically upregulated in IPF: **c** Insulin Growth Factor Binding Protein 5 (IGFBP-5). **d** Smooth muscle alpha-2 actin (ACTA-2). Examples of genes upregulated in both NSIP and normal controls: **e** Long Pentraxin 3 (PTX-3). **f** Indoleamine 2–3-dioxygenase-1 (IDO-1). Examples of genes specifically upregulated in NSIP: **g** Interferon-induced protein 44-like (IFI-44). H. LDL receptor related protein 2 (LRP-2)

SAM analysis showed that the genes with increased expression in IPF compared to NSIP were involved in epithelial-to-mesenchymal transition (*insulin growth factor binding protein-5* [IGFBP-5], *Prominin-1*), myofibroblast differentiation and proliferation (*smooth muscle alpha-actin* [ACTA-2], OPN), collagen deposition (OPN, IGFBP-5, POSTN), matrix remodeling (MMP-1, MMP-7, ACTA-2) peripheral blood mononuclear cells (PBMCs) proliferation and infiltration (IGFBP-5, MMP-7) and senescence (IGFBP-5, OPN, MMP-2). Most genes were also markedly upregulated in IPF when compared to normal controls. MMP-1 and OPN were the top 2 upregulated genes in IPF compared to controls (Additional file 3: Table S1). However, several genes previously studied in IPF (POSTN, OPN, MMP-1, MMP-7, Prominin-1) were also significantly upregulated in NSIP, when compared to controls (Fig. 2). In contrast, the increased expression of IGFBP-5 and 6, Mucin-5B, ACTA-2 was specific for IPF (Fig. 2). The expression of *surfactant protein-D* and *heme oxygenase-1* was decreased in IPF compared to both NSIP and controls, while the expression of VEGF-A was reduced in both IPF and NSIP, compared to normal controls. The expression of 3 biologically relevant genes (MMP-7, OPN, IGFBP-5) in IPF vs. NSIP was confirmed by RT-PCR (Additional file 4: Figure S3).

IPA demonstrated the involvement of relevant genes (OPN, POSTN, MMP-1, MMP-7, Prominin-1, and others) in the network “connective tissue disease, organismal injury and abnormality” (Additional file 5: Figure S4A), and other functions related to cell movement and connective tissue disorder (Additional file 3: Table S2). GSEA analysis (Table 4) demonstrated that numerous gene sets were significantly enriched in IPF vs. NSIP, mostly including biological functions related to cellular movement and proliferation.

**Table 4:** Top 5 gene-sets significantly enriched in the IPF group (GSEA)

Gene Set	NES	p value	q value
Axoneme assembly (biological process)	−2.65	< 0.001	< 0.001
Microtubule bundle formation (biological process)	−2.56	< 0.001	< 0.001
Ciliary plasm (cellular component)	−2.53	< 0.001	< 0.001
Cilium movement (biological process)	−2.44	< 0.001	< 0.001
Motile cilium (cellular component)	−2.44	< 0.001	< 0.001

The positive enrichment score indicated a correlation with the IPF group. Ontologies are indicated in brackets

### Gene expression profile of NSIP

Genes with significantly increased expression in NSIP, compared to both IPF and normal controls, were involved in regulatory mechanisms of immune reaction, including the alloreactive T cell response (*indoleamine 2–3-dioxygenase-1* [IDO-1]), the humoral arm of innate immunity (*long pentraxin-3* [PTX-3], *IFN-induced protein 44-like* [IFI-44]) and the recruitment of leukocytes into the lung compartment (*Endocan*, *LDL receptor-related protein-2* [LRP-2]). The top 25 genes upregulated in NSIP vs. controls are shown in Additional file 3: Table S3, which notably includes POSTN.

IPA demonstrated the involvement of relevant genes into pathways of interferon signaling, inflammatory response, granulocyte adhesion and anti-microbial response (Additional file 3: Table S4), and in the network “anti-microbial response, inflammatory response and cancer” (Additional file 5: Figure S4B). Similarly, GSEA demonstrated that gene sets significantly enriched in NSIP vs. IPF involved interferon-gamma-mediated signaling and production, and defense response to virus (Table 5).

**Table 5:** Top 5 gene-sets significantly enriched in the NSIP group (GSEA)

Gene Set	NES	p value	q value
Interferon-gamma-mediated signaling pathway (biological process)	2.48	< 0.001	< 0.001
Defense response to virus (biological process)	2.42	< 0.001	< 0.001
Response to type I Interferon (biological process)	2.30	< 0.001	< 0.001
Regulation of Interferon-gamma production (biological process)	2.27	< 0.001	< 0.001
Regulation of Interleukin-12 production (biological process)	2.26	< 0.001	0.005

*NES* Normalized Enrichment Score

q value: False Discovery Ratio

The positive enrichment score indicated a correlation with the NSIP group. Ontologies are indicated in brackets

### Unsupervised clustering analysis identifies an “intermediate” group of IPF patients

**Fig. 3:**
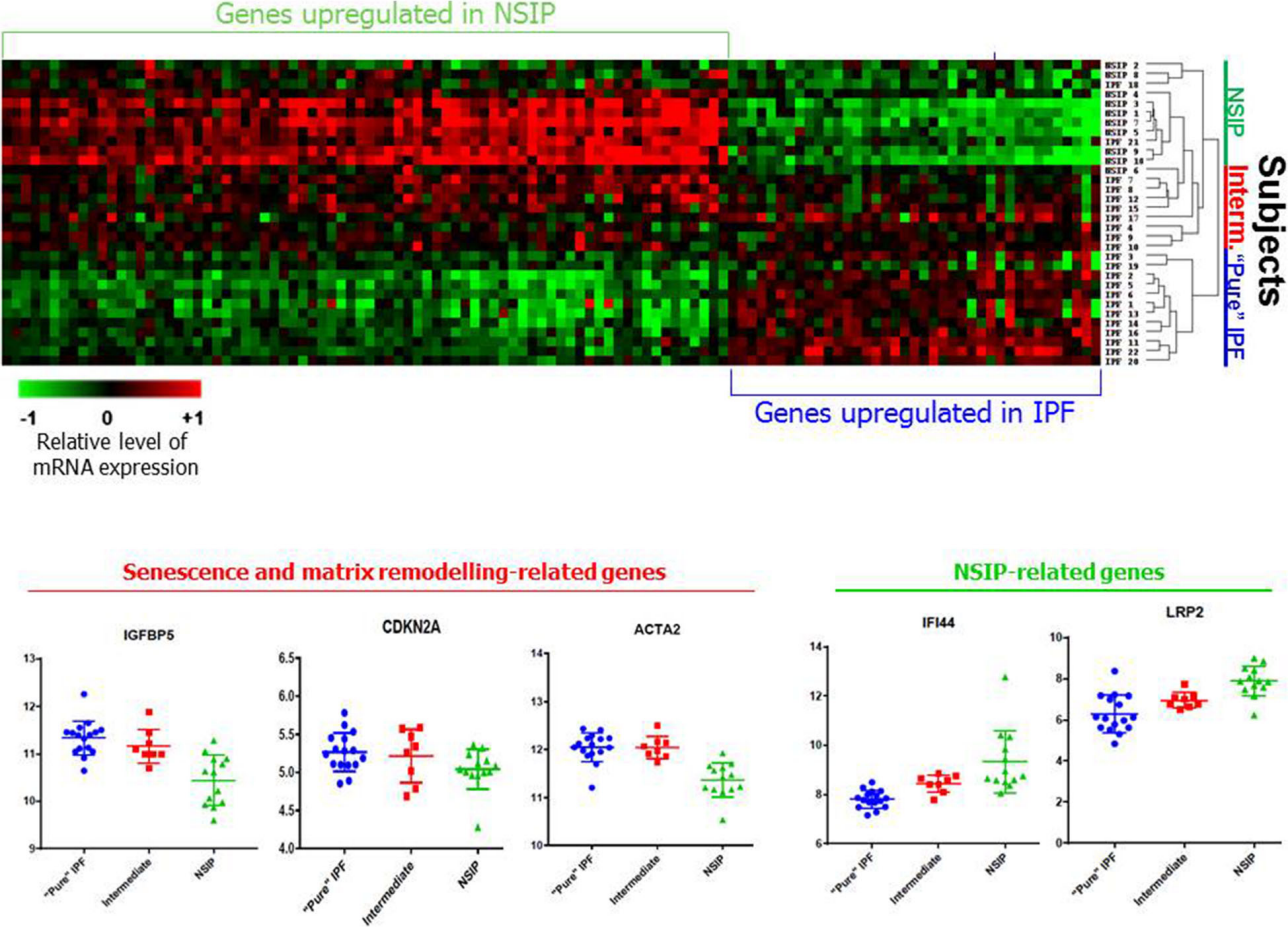
Heat map representing differentially expressed genes (fold change ≥1.50 and q value< 5%),) and hierarchic clustering. **a** Hierarchic clustering based on 146 differentially expressed genes (IPF vs. NSIP groups) in IPF (*n* = 22), NSIP (*n* = 10) and IPF-NSIP (*n* = 5) groups. Each row corresponds to an individual sample, and each column corresponds to an individual gene. Each square on the matrix represents the expression level of an individual gene in each sample, with red and green indicating gene expression levels above or below, respectively, compared with each other. While an IPF and a NSIP cluster are identifiable, an “unpaired” group of 7 IPF subjects and 2 NSIP patients showed intermediate expression and no clear clustering

Unsupervised hierarchical clustering based on differentially expressed genes demonstrated that a subgroup of 8 IPF subjects did not clearly group in either patient cluster, but rather had intermediate expression of selected genes (Fig. 3). The remaining IPF cases clustered together in a “pure” IPF group. NSIP cases, in contrast, showed a compact, homogenous clustering.

We examined the expression of disease-specific genes in the intermediate group, and found that IGFBP-5 level was similar to that of the “pure” IPF cluster, while in contrast, the expression of Mucin-5B was reduced to the same level observed in NSIP cases. LRP-2 and IFI-44, which are NSIP-specific, were also increased in the intermediate cluster, compared to the “pure” IPF group, to a similar expression level seen in NSIP group (Fig. 3). On the other hand, several senescence-related genes (IGFBP-5, p16) and matrix remodelling gene ACTA-2 were also increased in the intermediate group, compared to NSIP.

### The role of senescence

**Fig. 4:**
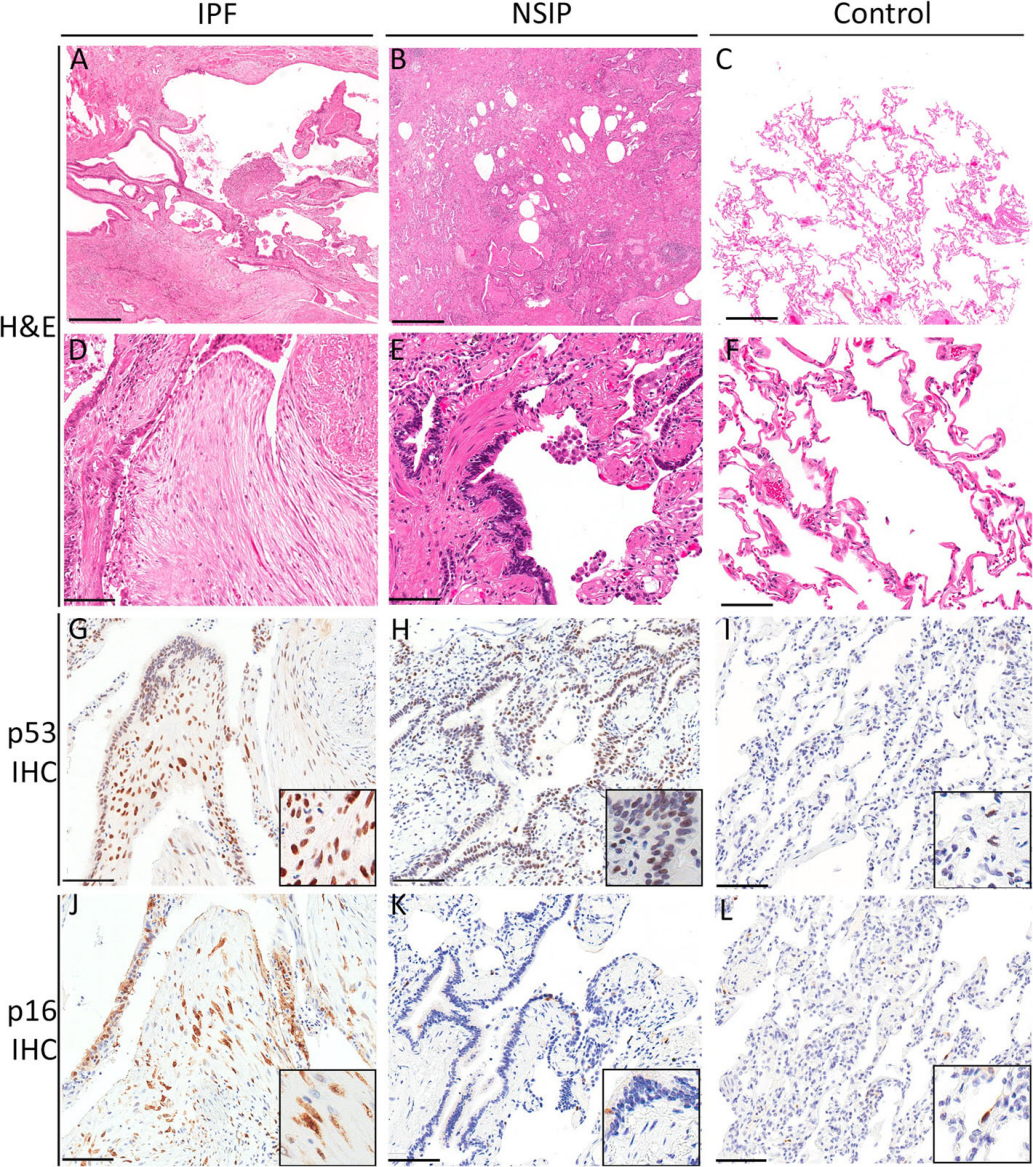
Immunohistochemistry studies. **a**-**f** Lower power (4X) and high power (20X) photomicrographs of hematoxylin and eosin stained sections of representative IPF (A,D), NSIP (B,E) cases and normal control lung tissue (C,F). **g**-**l** high power photomicrographs (20X) of immunohistochemistry for p53 and p16 of representative IPF (G,J), NSIP (H,K) cases and normal control lung tissue (I,L). Inserts show high power magnification. Scale bar for A-C = 500 μm, D-L = 100 μm

Given the increasingly recognized role of cellular senescence in IPF, we analyzed the expression of senescence effectors, senescence-associated secretory phenotype (SASP) growth factors and SASP matrix remodeling markers. Interestingly, the expression of most genes involved was not significantly increased in IPF (Additional file 3: Table S5). However, IGFBP-5, which is strongly and specifically upregulated in IPF (Fig. 2), plays an important role in the regulation of cellular senescence via a p53-dependent pathway [19]. p16, another key senescence effector, was modestly upregulated in IPF vs. NSIP (fold change 1.15) and, on IHC, 15 out of 23 IPF cases had positive p16 expression on fibroblasts. p16 staining was also diffusely noted on metaplastic epithelium (Fig. 4, Additional file 3: Table S6). In NSIP, p16 was focally but variably expressed in metaplastic epithelium, however no staining of fibroblasts was observed in the 13 cases examined (Fig. 4, Additional file 3: Table S6). IHC for POSTN and OPN did not distinguish IPF from NSIP (Additional file 3: Table S6).

### Serial periostin serum levels predict the clinical course of IPF

Senescence effector and matrix remodeling protein POSTN was among the top 10 upregulated genes in IPF, compared to normal controls (Additional file 3: Table S1A). Although a similar increase of gene expression was seen in NSIP vs. controls, POSTN serum levels were the only significant predictor of clinical progression in a cohort of patients with IPF, longitudinally followed for a period of 12 months. ROC analysis demonstrated that a POSTN level ≥ 338 ng/mL at baseline or its longitudinal increase over 338 ng/mL predicted clinical progression (area under the curve = 0.76, *p* = 0.0025) with 79% sensitivity and 73% specificity. Details on ROC analysis are shown in Additional file 3: Table S7. Univariate regression analysis (Table 6) confirmed POSTN serum levels predicted clinical progression significantly (H.R. 4.25, *p* = 0.0045). The serum levels of the other biomarkers investigated were not significant predictors of clinical progression (Table 6, Additional file 3: Table S7).

**Table 6:** Univariate regression analysis of biomarkers serum levels against 12-month clinical progression in a cohort of 34 patients with IPF

Biomarker	Hazard Ratio (C.I.)	*p* value
Periostin (> 338 ng/ml or increase > 338 from baseline)	1.00 (0.99–1.00) 4.25 (1.53–15.00)	0.0693 < 0.0001
Osteopontin	1.00 (0.99–1.00)	n.s.
KL-6	0.98 (0.76–1.25)	n.s.
MMP-9	1.00 (1.00–1.00)	n.s.
Surfactant protein A	1.00 (1.00–1.00)	n.s.
CCL-18	1.00 (0.99–1.02)	n.s.

*n.s.* not significant, *KL-6* Krebs von den Lungen-6, *MMP-9* Matrix Metalloproteinase 9, *CCL-18* C-C Motif Chemokine Ligand 18

## Discussion

Comprehensive genome-wide expression profiling identified gene upregulation, gene sets and pathways specific for either IPF or NSIP, but several genes that were previously thought to be specifically involved in IPF, were actually found to be equally or similarly upregulated in NSIP. Truly specific genes for IPF and NSIP, respectively, were however identified, including genes related to senescence in IPF only. IGFBP-5, a senescence-related growth factor, emerged as discriminator of IPF vs. NSIP. Unsupervised clustering analysis revealed the existence of a subgroup of IPF patients with only intermediate, ambiguous expression of selected genes. Translating results at the protein level, IHC analysis identified the expression of p16, a senescence marker, in fibroblasts as a potential diagnostic marker of IPF for clinical use, and serial serum levels of POSTN, a senescence matrix remodelling effector, as a predictor of disease progression.

The gene expression profile of NSIP received little attention [20, 21]. Yang et al. previously examined IPF and NSIP cases, but only half of cases were from large explant samples, both sporadic and familial cases were considered, and microarray analysis was limited to SAM [22]. Kim et al. analyzed a large amount of samples, but most consisted of surgical lung biopsies, rather than lung explants, and no normal controls were considered [20]. In our study, integrated SAM, IPA and GSEA analyses in NSIP pointed to alloreactive T cell response, the humoral arm of innate immunity, IL-12 production regulation, and recruitment of leukocytes into the lung compartment and granulocyte adhesion as main processes involved in NSIP. These, importantly, are all part of anti-microbial response via the IFN-gamma signaling pathway. IFN-gamma is distinguished from other interferons by its ability to coordinate the transition from innate immunity to adaptive immunity [23], but its substantial contribution to T cell differentiation and immunoglobulin class switching in B cells underlines also a decisive role in adaptive immune responses in autoimmunity [24]. In regards to specific markers of NSIP, in comparison to both IPF and normal controls, we identified IFI-44, involved in inflammation and innate immune response pathways [25], and LRP-2 (also known as megalin), a regulator of protein leak during lung injury, highly expressed on the apical surface of epithelial cells [26], as 2 potential candidates, which will require further clinical confirmation.

Notably, in this study, the histologic definition of NSIP was based on whole explanted lungs, not normally available in clinical practice, with well recognizable histopathologic pattern, far from a generic definition of “end-stage pulmonary fibrosis”. The finding of a gene expression profile well distinguished from that of IPF is important, as it reaffirms the existence of NSIP as a stand-alone condition that needs better clinical characterization. While PCA analysis showed a degree of overlap across the genome between IPF and NSIP, which could be explained by our subsequent clustering analysis findings, pathways and functional analysis revealed a radically different and remarkably homogenous gene signature in NSIP.

The gene signature of IPF has been reassuringly reproducible across several microarray studies [27–29]. Our findings confirm epithelial-to-mesenchymal transition, myofibroblasts proliferation, collagen deposition and PBMCs recruitment and infiltration as leading mechanisms of IPF at the transcriptional level, with the important addition of senescence. However, another novel finding of this study was the complete lack of specificity in the upregulation of POSTN, OPN, MMP-1, MMP-7 and PROM-1, previously and extensively studied in IPF, but not considered in NSIP. Not surprisingly, we found that IHC for POSTN, OPN and, as shown previously by Huh et al. [30], MMP-7 does not distinguish IPF from NSIP. However, by cross-checking upregulated genes against both NSIP and normal controls, we were still able to identify a gene signature truly specific for IPF, which includes MUC5B, ACTA-2 and IGFBP-5. The latter is a critical trigger of senescence [31], which showed a nearly complete separation between IPF and NSIP groups, with differential expression confirmed by RT-PCR.

Based on this finding and on recent literature [32, 33], we specifically looked at senescence as a mechanism considered relevant into the pathogenesis of IPF. Senescence is a state of irreversible replicative arrest induced by pro-ageing stressors, associated with resistance to apoptosis and secretion of SASP [32]. This includes matrix remodelling proteases and growth factors, cytokines and chemokines. Senescence is not always necessarily detrimental, as it can for example protect from cancer by disabling cells accumulating potentially deleterious damage and mutations [34]. However, senescence-promoted secretome and the lack of clearance of senescent fibroblasts are indeed fibrogenic (32,33). Although we found relatively few senescence-related and SASP genes to be upregulated in IPF vs. either NSIP or controls, when considering homogenized, whole lung tissue samples, the senescent signal on epithelial cells may be diluted. Furthermore, senescence in IPF occurs prevalently in epithelial cells [35], as confirmed on IHC studies, and these may not account for much of the overall gene expression signal in homogenized samples.

The occurrence of senescence in cells playing a key biological role in the pathogenesis of fibrotic lung disease might be more important that the overall extent of this process in the lung tissue. Among senescence-related genes, p16 is a cyclin-dependent kinase inhibitor that blocks cell cycle progression by antagonizing cyclin-dependent kinases [36]. The CDKN2A gene (which encodes p16) was only modestly upregulated in IPF compared to NSIP, but, importantly, p16 immunostaining on fibroblastic foci differentiated most, although not all, IPF cases from NSIP. Metaplastic bronchial epithelium overlying fibroblastic foci also often expresses high levels of p16. This could be induced in a paracrine fashion by the activity of IGFBP-5 via a p53-dependent mechanism [19]. While we should consider that no single marker may define a cell as senescent, since none of these markers are exclusive to cellular senescence [34], p16 immunostaining as a potential discriminator in IIP will deserve further studies.

Another component of SASP, which may turn senescent fibroblasts into proinflammatory cells [37], is POSTN, a matrix-remodelling protein. Naik et al. previously found that baseline POSTN levels were predictive of clinical progression [38]. POSTN is one of the most strongly upregulated genes in IPF, although not specifically for this condition. Consistently, Ohta et al. did not find significant differences in POSTN levels between IPF and NSIP subjects [39]. We considered longitudinal changes of POSTN in IPF patients and found that its increase predicts clinical progression better than other biomarkers considered, outperforming a panel of alternative biomarkers selected from literature review. These results will need to be confirmed in a larger population of patients with IIP.

Unsupervised clustering analysis based on differentially expressed genes, further verified against normal controls, provided important information, revealing significant transcriptional heterogeneity of IPF, which is striking, considering the radiographic-pathologic homogeneity of the cohort we selected. In contrast, NSIP appears to be a much more transcriptionally homogenous condition. Alongside a homogenous and compact NSIP group and a “pure” IPF group, an intermediate group of IPF subjects with ambiguous expression did not clearly cluster with either entity. Intriguingly, the intermediate group of patients expressed high levels of 2 NSIP-specific markers (LRP-2 and IFI-44), on one hand, and of a combination of senescence and matrix remodelling-related genes (IGFBP-5, CDKN2A [encoding p16], ACTA-2) on the other. The existence of this “intermediate” cluster of IPF patients, expressing both NSIP-related and SASP-related genes, cannot be attributed to a mixed histologic pattern, as pathologic diagnosis was confirmed on whole lung explants. Furthermore, all IPF cases in this study featured an entirely typical HRCT pattern of UIP.

Considering the interesting observations of radiographic “evolution” of biopsy-proven NSIP cases into an UIP/IPF pattern [40], and the consistently younger age of NSIP patients compared to IPF patients, we advance the hypothesis that “senescent” NSIP itself may represent a risk factor to develop superimposed IPF. Cases with intermediate gene expression pattern, with an upregulation of both NSIP-specific and senescence-related genes, may represent an occurred or still occurring “transition” from NSIP to IPF, possibly driven by senescence. Such hypothesis will need much stronger evidence to be confirmed, but it would help to explain the clinical variability observed not only in IPF, but even in NSIP: while many cases of NSIP respond to immunosuppressive therapy and survive for a long time, some stop responding and need to be referred to LTx. An alternative explanation for these findings would be that a subset of patients with IPF exhibits an alternative inflammatory gene expression profile, resembling the transcriptional profile of NSIP, reaffirming the existence of significant variability in the spectrum of this disease. However, this would leave the clinical variability of NSIP unexplained, given the transcriptional homogeneity we observed in this entity.

## Conclusions

Comprehensive gene expression profiling from whole lung explants aligns separate, well-recognizable histopathologic patterns of IIP with distinct transcriptional profiles and differentially expressed genes, and does not support the notion of NSIP as an early manifestation of IPF, but rather point to a standalone, transcriptionally homogenous condition, even in advanced fibrotic cases requiring LTx. LRP-2 and IFI-44 were identified as candidate molecular markers of NSIP, further validated against normal controls. This, however, does not exclude that “senescent” NSIP itself may represent a risk factor for developing IPF, given the remarkable degree of transcriptional overlap observed in a subgroup of IPF cases. Senescence-related genes are indeed prominent in IPF: we identified IGFBP-5 as a candidate molecular marker specific for IPF; p16 as a candidate fibroblast protein marker of IPF; and POSTN serum levels as better predictors of clinical progression, although not specific for IPF. These findings will likely stimulate validation studies in smaller, routinely available biopsy samples, as well as in larger populations.

## Additional files

Additional file 1: **Figure S1. A**. Probe intensity histogram. B. F ratio: signal-to-noise ratio, IPF vs. NSIP analysis across the whole genome. The bar indicates the average signal for all genes. The height of the bar is the mean square. UIP=IPF (JPG 57 kb)

Additional file 2: **Figure S2.** Principal component analysis, a global analysis across the whole genome. IPF and NSIP groups are shown. UIP=IPF. (JPG 114 kb)

Additional file 3: **Table S1.** Top 25 upregulated genes in IPF vs. control (SAM analysis). **Table S2.** IPF vs. control, Ingenuity Pathway Analysis (IPA). In IPA, “functions” are divided in 3 categories: “disease and disorders”, “molecular and cellular functions” and “physiological system development and function”. **Table S3.** Top 25 upregulated genes in NSIP vs. control (SAM analysis). **Table S4.** NSIP vs. control, Ingenuity Pathway Analysis (IPA). In IPA, “functions” are divided in 3 categories: “disease and disorders”, “molecular and cellular functions” and “physiological system development and function”. **Table S5.** Gene expression of cellular senescence biomarkers in IPF vs. NSIP and vs. normal controls. Significantly upregulated genes are indicated with *. Pro- and anti-fibrotic properties of metalloproteinases (MMPs) are indicated in brackets under the gene name. **Table S6.** Summary of immunohistochemistry findings in IPF and NSIP. **Table S7.** Receiver operating characteristic (ROC) analysis of serum biomarkers vs. 12-month clinical progression. (DOCX 47 kb)

Additional file 4: **Figure S3.** Comparison of gene expression levels determined by oligonucleotide microarray (left) and by quantitative RT-PCR (right; ratio with the expression the housekeeping gene GADPH) in the IPF and NSIP groups: A. MMP-7. B: OPN. C. IGFBP-5. (JPG 81 kb)

Additional file 5: **Figure S4.** Ingenuity network analysis, networks with highest scores. Networks were scored based on the number of network eligible molecules they contained. Network eligible molecules with relatively increased expression are shown in red, whereas molecules with relatively reduced expression are shown in green. The intensity of the color is proportional to the fold change. Non-colored nodes represent genes added by Ingenuity pathway analysis based on its network algorithm but not upregulated in the actual microarray data. A. Network “Connective tissue disease, organismal injury and abnormality, cancer” (score = 29). Network eligible molecules with relatively increased expression in the IPF group are shown in red, whereas molecules with relatively increased expression in the NSIP group are shown in green. B. Network “Anti-microbial response, inflammatory response and cancer” (score = 42). Network eligible molecules with relatively increased expression I the NSIP group are shown in red. (JPG 53 kb)

## Abbreviations

ACTA-2: Smooth muscle alpha-actin
CCL-18: Chemokine-(C-C motif)ligand-18
GSEA: Gene set enrichment analysis
HRCT: High-resolution chest CT scan
IDO-1: Indoleamine 2–3-dioxygenase-1
IFN: Induced protein 44-like [IFI-44]
IGFBP-5: Insulin growth factor binding protein-5
IHC: Immunohistochemistry
IIP: Idiopathic interstitial pneumonia
ILD: Interstitial lung disease
IPF: Idiopathic pulmonary fibrosis
KL-6: Krebs von den Lungen-6
LRP-2: LDL receptor-related protein-2
MMP-9: Matrix metallopeptidase 9
NSIP: Non-specific interstitial pneumonia
OPN: Osteopontin
PBMCs: Peripheral blood mononuclear cells
POSTN: Periostin
PTX-3: Long pentraxin 3
SAM: Significance analysis of microarray
SASP: Senescence-associated secretory phenotype
SP-A: Surfactant protein A
UIP: Usual interstitial pneumonia
