# Supplementary figures and images for: Comprehensive gene expression profiling identifies distinct and overlapping transcriptional profiles in non-specific interstitial pneumonia and idiopathic pulmonary fibrosis
Source: Respir Res. 2018 Aug 15;19:153. doi: 10.1186/s12931-018-0857-1 (PMC6094889; doi:10.1186/s12931-018-0857-1)

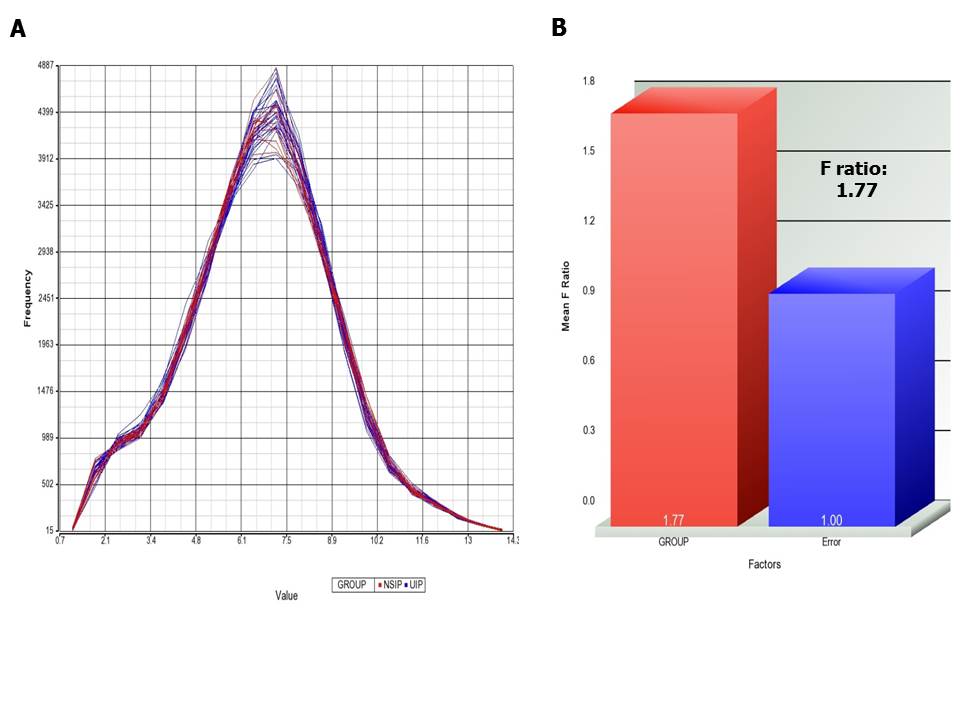

Supplement: Supplementary file 1 — Figure S1. A. Probe intensity histogram. B. F ratio: signal-to-noise ratio, IPF vs. NSIP analysis across the whole genome. The bar indicates the average signal for all genes. The height of the bar is the mean square. UIP=IPF (JPG 57 kb) [file 12931_2018_857_MOESM1_ESM.jpg]

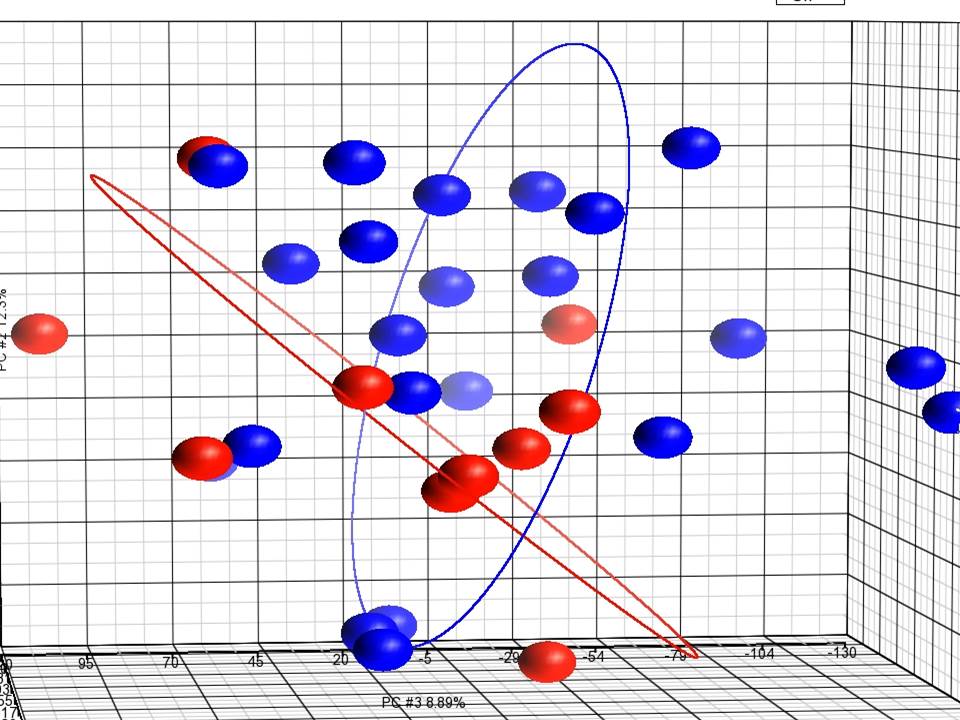

Supplement: Supplementary file 2 — Figure S2. Principal component analysis, a global analysis across the whole genome. IPF and NSIP groups are shown. UIP=IPF. (JPG 114 kb) [file 12931_2018_857_MOESM2_ESM.jpg]

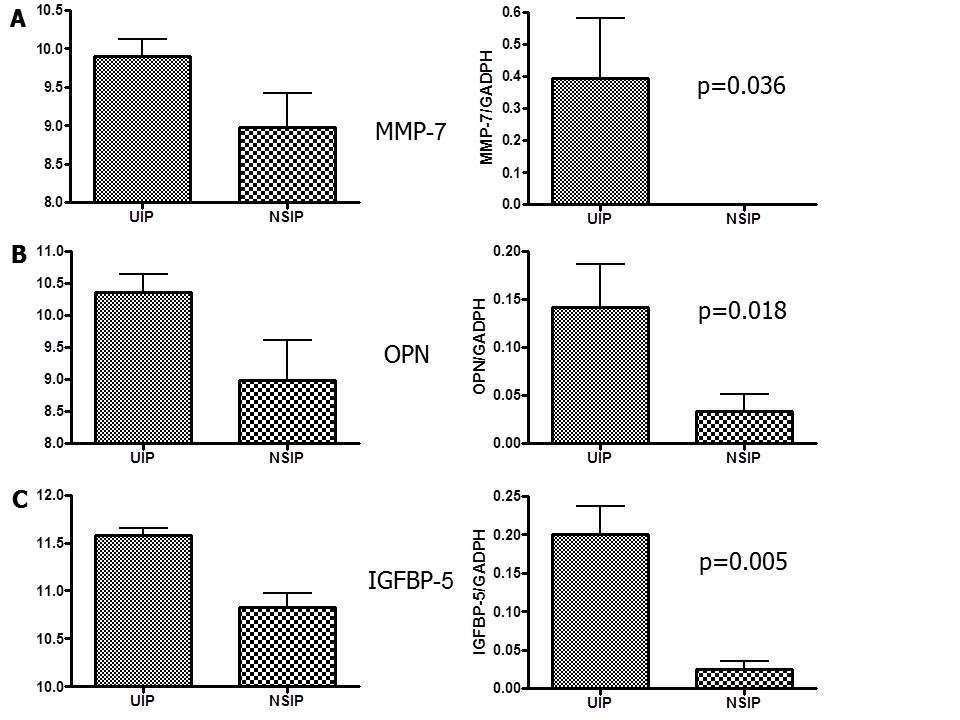

Supplement: Supplementary file 4 — Figure S3. Comparison of gene expression levels determined by oligonucleotide microarray (left) and by quantitative RT-PCR (right; ratio with the expression the housekeeping gene GADPH) in the IPF and NSIP groups: A. MMP-7. B: OPN. C. IGFBP-5. (JPG 81 kb) [file 12931_2018_857_MOESM4_ESM.jpg]

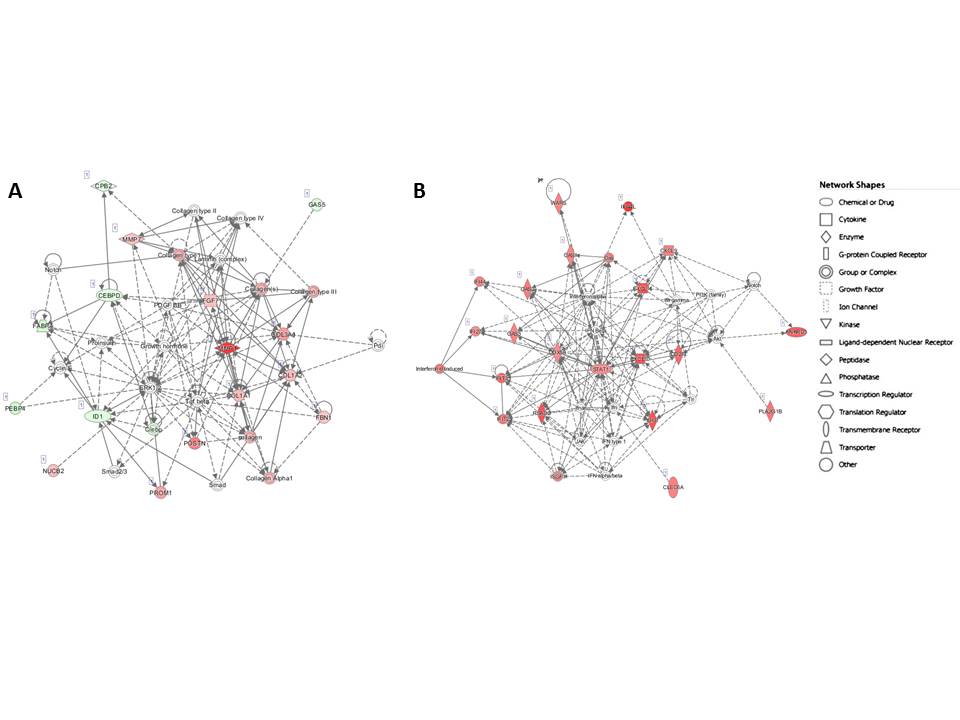

Supplement: Supplementary file 5 — Figure S4. Ingenuity network analysis, networks with highest scores. Networks were scored based on the number of network eligible molecules they contained. Network eligible molecules with relatively increased expression are shown in red, whereas molecules with relatively reduced expression are shown in green. The intensity of the color is proportional to the fold change. Non-colored nodes represent genes added by Ingenuity pathway analysis based on its network algorithm but not upregulated in the actual microarray data. A. Network “Connective tissue disease, organismal injury and abnormality, cancer” (score = 29). Network eligible molecules with relatively increased expression in the IPF group are shown in red, whereas molecules with relatively increased expression in the NSIP group are shown in green. B. Network “Anti-microbial response, inflammatory response and cancer” (score = 42). Network eligible molecules with relatively increased expression I the NSIP group are shown in red. (JPG 53 kb) [file 12931_2018_857_MOESM5_ESM.jpg]
