# Supplementary material for: Comprehensive gene expression profiling identifies distinct and overlapping transcriptional profiles in non-specific interstitial pneumonia and idiopathic pulmonary fibrosis
Source: Respir Res. 2018 Aug 15;19:153. doi: 10.1186/s12931-018-0857-1 (PMC6094889; doi:10.1186/s12931-018-0857-1)
Supplement: Supplementary file 3 — Table S1. Top 25 upregulated genes in IPF vs. control (SAM analysis). Table S2. IPF vs. control, Ingenuity Pathway Analysis (IPA). In IPA, “functions” are divided in 3 categories: “disease and disorders”, “molecular and cellular functions” and “physiological system development and function”. Table S3. Top 25 upregulated genes in NSIP vs. control (SAM analysis). Table S4. NSIP vs. control, Ingenuity Pathway Analysis (IPA). In IPA, “functions” are divided in 3 categories: “disease and disorders”, “molecular and cellular functions” and “physiological system development and function”. Table S5. Gene expression of cellular senescence biomarkers in IPF vs. NSIP and vs. normal controls. Significantly upregulated genes are indicated with *. Pro- and anti-fibrotic properties of metalloproteinases (MMPs) are indicated in brackets under the gene name. Table S6. Summary of immunohistochemistry findings in IPF and NSIP. Table S7. Receiver operating characteristic (ROC) analysis of serum biomarkers vs. 12-month clinical progression. (DOCX 47 kb) [file 12931_2018_857_MOESM3_ESM.docx]

Comprehensive gene expression profiling identifies distinct and overlapping transcriptional profiles in non-specific interstitial pneumonia and idiopathic pulmonary fibrosis

Matthew J. Cecchini^1^, Karishma Hosein^2^, Christopher J. Howlett^1^, Mariamma Joseph^1^, Marco Mura^2,3^

^1^Department of Pathology, Western University, London, Canada

^2^Division of Respirology, Western University, London, Canada

^3^Toronto Lung Transplant Program, University of Toronto, Toronto, Ontario

**SUPPLEMENTAL MATERIAL**

**MATERIAL AND METHODS**

# Subjects

Specimens were obtained from the peripheral area at the base of each organ as soon as the first recipient lung was taken out, snap frozen in liquid nitrogen, and stored at -80ºC.

Intraoperative mean PAP (mPAP) was calculated according to the following formula: diastolic PAP + 1/3(systolic PAP–diastolic PAP). Pulmonary capillary wedge pressure (PCWP) was not recorded pre-operatively.

For histologic examination, representative 4 µm thick sections were taken from each lobe of the each explanted lung and additional sections were taken from the peribronchial lymphnodes. The final pathologic diagnosis was reached by consensus by at least 2 lung pathologists.

**RNA extraction and cDNA synthesis**

RNA was isolated with Trizol® Reagent (Invitrogen Canada Inc., Burlington, ON); a clean up step was performed then with RNeasy MinElute Cleanup kit (Qiagen Canada Inc., Mississauga, ON). At least 50 µl of RNA were collected from each sample and divided into two parts: 10 µl were used for RNA qualification and microarray; 40 µl were used for subsequent assays. RNA was qualified by RNA nano chips on an Agilent Bioanalyzer 2100 (Agilent Technologies, Inc., Santa Clara, CA) and the RNA Integrity Number was determined as a measure of the quality of the RNA. An RNA integrity number of 7.0 was taken as acceptable to proceed to the microarray analysis. cDNA was synthesized in 80 µl from 4 µg of RNA with High-Capacity cDNA Reverse Transcription kits (Applied Biosystems, Carlsbad, CA, Cat No. 4374966). cDNA-synthesis was carried out on a PTC-100™ Programmable Thermal controller (MJ Research Inc., Waltham, MA), at 25ºC for 10 min, 37ºC for 120 min, 85ºC for 5 min, 4ºC. RNA was qualified by RNA nano chips on an Agilent 2100 Bilanalyzer (Agilent Technologies, Santa Clara, CA). Microarray was performed with Genechip® Human Gene 1.0 ST on an Affymetrix Genechip Scanner 3000 and Genechip® Fluidics Station 450 (Affymetrix). Annotation of probe sets was performed using Expression Console software. Entrez Gene ID ([www.ncbi.nlm.nih.gov](http://www.ncbi.nlm.nih.gov)) was used to filter out probe sets not yet confirmed to be genes.

**Microarray Data Analysis**

Affymetrix CEL files were imported into the Partek software (Partek Inc., St. Louis, MO) for the preliminary analysis. Probe-level data were pre-processed. This included robust multi-array average (Rma) background correction and data normalization, which was performed across all arrays using quantile normalization. Background-adjusted, normalized values were then compiled, or summarized, using the median polish technique, to generate a single measure of expression. As an exploratory data analysis, Principal Component Analysis (PCA) mapping and F Ratio analysis (signal-to-noise ratio) were also performed.

For Significant Analysis of Microarray (SAM), (<http://www-stat.stanford.edu/~tibs/SAM>) 33,297 probe sets were used. The q value, which was adopted as a multiple comparison correction and used to identify differentially expressed genes, represents the minimal false discovery ratio (FDR) at which an individual hypothesis test may be called significant.

For the Pathways/Networks Discovery Analysis we used Ingenuity Pathway Analysis (IPA) (Ingenuity systems Inc., Redwood City, CA); the identifier type was GenBank. One hundred forty six differentially expressed genes selected by SAM analysis were included in the Ingenuity Core Analysis, which allows interpreting large microarray dataset in the context of biological processes, pathways and molecular networks. The main expression value was the fold change (expression in the IPF group or NSIP group vs. normal controls), and the secondary expression value was the q value. A stringent filter for human molecules and relationships was used. For Function analysis, the p value (Fisher Exact Test) was calculated considering the number of functional analysis molecules that participate in that function and the total number of molecules that are known to be associated with that function in Ingenuity’s knowledge base. For the Network analysis, the score was used to rank networks according to their degree of relevance to the Network Eligible molecules in the dataset. The score (Fisher’s Exact Test) takes into account the number of Network Eligible molecules analyzed and the total number of molecules in Ingenuity’s knowledge base. The score is the negative log of the p value calculated with Fisher’s Exact Test and is a measure of the number of Network Eligible Molecules in a network. Therefore, networks were scored based on the number of Network eligible molecules they contain.

Gene-Set Enrichment Analysis (GSEA) ([http://www.broadinstitute.org/gsea](http://www.broadinstitute.org/gsea/)) is a computational method that determines whether an *a priori* defined set of genes shows statistically significant, concordant differences between two biological states. GSEA was used to determine whether members of a given gene set *S* tend to occur primarily toward the top (or bottom) of the list *L*, in which case the gene set is correlated with the phenotype class distinction, or are randomly distributed. By default, the ranking metric is the signal-to-noise ratio. The enrichment score (ES) reflects the degree to which a gene set is overexpressed at the top or bottom of a ranked list of genes. The normalized ES (NES) takes into account for differences in gene set size and in correlations between gene sets and the expression dataset. C5 Gene Ontology was used as gene set database. Each GO term belongs to one of the three ontologies: molecular function, cellular component or biological process.

For hierarchic clustering, Cluster 3.0 and Treeview (Eisen’s Laboratory, Stanford University) were used (<http://rana.lbl.gov/eisen/?page_id=7>). Unsupervised clustering does not take any of the experimental variables such as treatment, phenotype, tissue, etc. into account while clustering.

#### Quantitative Real-Time RT-PCR

Three genes (MMP-7, Osteopontin, IGFBP-5), based on microarray profiles of the IPF vs. NSIP analysis, were tested on an RT-PCR platform for verification in the same development set samples. Ribosomal protein large P0 (RPLP0) was used as housekeeping gene. All target genes and housekeeping gene primers were designed by ABI Prism® Primer Express™ Version 2.0 (Applied Biosystems). A special DNA fragment was created, including all target and housekeeping gene as RT-PCR standard. The DNA fragment carried out by a 50 μl regular PCR on a PTC-100™ Programmable Thermal controller (MJ research Inc.), at 94ºC for 2 min, (94ºC 30 s, 59ºC 45 s, 72ºC 1 m 15s) X 40 cycles, 72ºC for 10 min. Regular PCRs were performed by 10 X High Fidelity PCR buffer (Invitrogen, Cat. n.52045), Platinum® Taq polymerase High Fidelity (Invitrogen, Cat no. 11304-029), 50 mM MgSO_4_ (Invitrogen, Cat no. 52044), 10 mM dNTP Mix (Invitrogen, Cat no. 100004893). A SYBR GREEN real time PCR was performed on ABI PRISM 7900HT system. PCR was composed of Power SYBR^®^ GREEN PCR 2X Master Mix (Applied Biosystems, Cat. No. 4367659), 200 nM primer and 2 μl 500 ng/μl cDNA, at 50ºC for 2 min, 95ºC for 10 min, (95ºC for 15s ec, 60ºC for 1 min) X 40 cycles.

# Immunohistochemistry

All slides and immunohistochemistry were reviewed by at least 2 pathologists (MJC, CH, MGJ). Immunohistochemistry for p53 and p16 was performed using standard techniques on the DAKO autostainer platform with the p16 mouse monoclonal antibody E6H4 (Roche, Tucson, AZ) and the p53 mouse monoclonal antibody DO-7 (DAKO, Santa Clara, CA). Periostin (PN) was manually stained using the goat polyclonal antibody S-15 antibody (Santa Cruz Biotechnology, Santa Cruz, Ca) at a 1/200 dilution and detected using the ImmPress AntiGoat Ig (Vector laboratories) and the DAB Substrate kit (Vector Laboratories).

**Statistical analysis**

All values will be expressed as mean ± standard deviation. The Kolmogorov-Smirnov test was used to test the normality of the distribution for each variable. For comparison of two separate groups either the unpaired *t*-test or the Mann-Whitney test was used, when indicated. P values <0.05 were regarded as significant. Cox proportional hazards regression analysis was used to identify significant serum markers predicting clinical progression of IPF. Receiver operating characteristics (ROC) analysis was used to determine the sensitivity and specificity of serum biomarkers in predicting clinical progression (c-statistics). The Prism 4 software package (GraphPad Software Inc., La Jolla, CA) was used for statistical analyses.

**SUPPLEMENTAL TABLES**

**Table S1**. Top 25 upregulated genes in IPF vs. control (SAM analysis).

| **NCBI Gene Symbol** | **NCBI Gene name** | **d** | **Fold change vs. NSIP group** | **q value** |
| --- | --- | --- | --- | --- |
| MMP1 | Matrix metallopeptidase 1 (interstitial collagenase) | 10.0 | 16.91 | <0.001 |
| SSP1 | Secreted phosphoprotein 1 (osteopontin) | 9.4 | 15.57 | <0.001 |
| BPIFB1 | BPI fold containing family B, member 1 | 5.0 | 14.38 | <0.001 |
| CP | Ceruloplasmin (ferroxidase) | 7.9 | 13.20 | <0.001 |
| ND6 | NADH dehydrogenase, subunit 6 (complex I) | 30.1 | 11.86 | <0.001 |
| POSTN | Periostin, osteoblast specific factor | 12.9 | 10.51 | <0.001 |
| DSC3 | Desmocollin 3 | 8.7 | 10.14 | <0.001 |
| DDX3Y | DEAD (Asp-Glu-Ala-Asp) box polypeptide 3, Y-linked | 4.0 | 9.65 | <0.001 |
| DNAH12 | dynein, axonemal, heavy chain 12 | 6.1 | 8.92 | <0.001 |
| CD163 | CD163 molecule | 16.9 | 8.86 | <0.001 |
| COL3A1 | Collagen, type III, alpha 1 | 20.1 | 8.42 | <0.001 |
| DYNC2H1 | Dynein, cytoplasmic 2, heavy chain 1 | 14.4 | 8.41 | <0.001 |
| PROM1 | Prominin 1 | 5.3 | 8.00 | <0.001 |
| DNAH12 | Dynein, axonemal, heavy chain 12 | 5.4 | 7.67 | <0.001 |
| SNORD114-2 | Small nucleolar RNA, C/D box 114-2 | 9.3 | 7.5 | <0.001 |
| TMF1 | TATA element modulatory factor 1 | 21.6 | 7.29 | <0.001 |
| EIF1AY | Eukaryotic translation initiation factor 1A, Y-linked | 4.1 | 7.08 | <0.001 |
| KRT5 | Keratin 5, type II | 6.5 | 7.05 | <0.001 |
| ZBBX | Zinc finger, B-box domain containing | 4.9 | 7.04 | <0.001 |
| PDCD4 | Programmed cell death 4 (neoplastic transformation inhibitor) | 24.5 | 7.04 | <0.001 |
| LUC7L3 | LUC7-like 3 pre-mRNA splicing factor | 21.0 | 6.76 | <0.001 |
| ZNF841 | Zinc finger protein 841 | 18.9 | 6.63 | <0.001 |
| CFH | Complement factor H | 17.4 | 6.60 | <0.001 |
| DNAH6 | Dynein, axonemal, heavy chain 6 | 5.86 | 6.49 | <0.001 |
| ESF1 | ESF1 nucleolar pre-rRNA processing protein homolog | 20.3 | 6.42 | <0.001 |

d = standardized change in expression (relative difference); q value= false discovery rate

**Table S2**. IPF vs. control, Ingenuity Pathway Analysis (IPA). In IPA, “functions” are divided in 3 categories: “disease and disorders”, “molecular and cellular functions” and “physiological system development and function”.

| **Name** | N. of molecules |
| --- | --- |
| **Disease and Disoders** | |
| Cancer | 76 |
| Organismal injury and abnormalities | 76 |
| Reproductive sustem disease | 60 |
| Connective tissue disorders | 17 |
| Gastrointestinal disease | 70 |
| **Molecular and cellular functions** | |
| Cellular assembly and organization | 24 |
| Cellular function and maintenance | 23 |
| Carbohydrate metabolism | 6 |
| Cell cycle | 4 |
| Cellular Development | 7 |
| **Physiological system development and function** | |
| Cardiovascular system development and function | 15 |
| Tissue morphology | 12 |
| Organismal functions | 7 |
| Hematological system development and function | 4 |
| Connective tissue development and function | 8 |

**Table S3**. Top 25 upregulated genes in NSIP vs. control (SAM analysis).

| **NCBI Gene Symbol** | **NCBI Gene name** | **d** | **Fold change vs. NSIP group** | **q value** |
| --- | --- | --- | --- | --- |
| CD163 | CD163 molecule | 15.1 | 11.5 | <0.001 |
| ND6 | NADH dehydrogenase, subunit 6 | 27.9 | 10.5 | <0.001 |
| SNORD114-2 | small nucleolar RNA, C/D box 114-2 | 8.4 | 8.28 | <0.001 |
| ESF1 | ESF1 nucleolar pre-rRNA processing protein homolog | 17.8 | 8.26 | <0.001 |
| CP | Ceruloplasmin (ferroxidase) | 5.3 | 8.2 | <0.001 |
| TMF1 | TATA element modulatory factor 1 | 16.6 | 7.9 | <0.001 |
| ZNF841 | zinc finger protein 841 | 14.5 | 7.1 | <0.001 |
| ZNF845 | zinc finger protein 845 | 12.8 | 7.1 | <0.001 |
| BIRC3 | Baculoviral IAP repeat-containing 3 | 8.7 | 6.9 | <0.001 |
| PI15 | Peptidase inhibitor 15 | 5.9 | 6.9 | <0.001 |
| DYNC2H1 | Dynein, cytoplasmic 2, heavy chain 1 | 11.8 | 6.8 | <0.001 |
| GCC2 | GRIP and coiled-coil domain containing 2 | 13.5 | 6.8 | <0.001 |
| POSTN | Periostin, osteoblast specific factor | 5.7 | 6.7 | <0.001 |
| EIF4A2 | eukaryotic translation initiation factor 4A2 | 15.1 | 6.7 | <0.001 |
| MPHOSPH10 | M-phase phosphoprotein 10 | 19.5 | 6.6 | <0.001 |
| COL3A1 | collagen, type III, alpha 1 | 10.3 | 6.5 | <0.001 |
| LUC7L3 | LUC7-like 3 pre-mRNA splicing factor | 15.3 | 6.4 | <0.001 |
| USP47 | ubiquitin specific peptidase 47 | 15.1 | 6.4 | <0.001 |
| DDX21 | DEAD (Asp-Glu-Ala-Asp) box helicase 21 | 9.4 | 6.4 | <0.001 |
| CCDC186 | coiled-coil domain containing 186 | 13.3 | 6.4 | <0.001 |
| PNN | pinin, desmosome associated protein | 17.5 | 6.3 | <0.001 |
| IVNS1ABP | influenza virus NS1A binding protein | 11.5 | 6.3 | <0.001 |
| TARS | threonyl-tRNA synthetase | 14.9 | 6.3 | <0.001 |
| IFT80 | intraflagellar transport 80 | 17.1 | 6.3 | <0.001 |
| SNORD78 | small nucleolar RNA, C/D box 78 | 11.6 | 6.2 | <0.001 |

d = standardized change in expression (relative difference); q value= false discovery rate

**Table S4**. NSIP vs. control, Ingenuity Pathway Analysis (IPA). In IPA, “functions” are divided in 3 categories: “disease and disorders”, “molecular and cellular functions” and “physiological system development and function”.

| **Name** | N. of molecules |
| --- | --- |
| **Disease and Disoders** | |
| Cancer | 69 |
| Endocrine system disorders | 33 |
| Organismal injury and abnormalities | 69 |
| Reproductive system disease | 51 |
| Gastrointestinal disease | 65 |
| **Molecular and cellular functions** | |
| Cell cycle | 6 |
| RNA post-transcriptional modification | 7 |
| Gene expression | 6 |
| Protein synthesis | 8 |
| Cellular compromise | 6 |
| **Physiological system development and function** | |
| Hematological system development and function | 4 |
| Digestive system development and function | 7 |
| Organismal development | 15 |
| Tissue morphology | 6 |
| Connective tissue development and function | 6 |

**Table S5**. Gene expression of cellular senescence biomarkers in IPF vs. NSIP and vs. normal controls. Significantly upregulated genes are indicated with *. Pro- and anti-fibrotic properties of metalloproteinases (MMPs) are indicated in brackets under the gene name.

| **NCBI Gene Symbol** | **NCBI Gene name** | **Fold change**  **vs. NSIP group**  **(q value)** | **D** | **Fold change**  **vs. control group**  **(q value)** | **D** |
| --- | --- | --- | --- | --- | --- |
| **SENESCENCE EFFECTORS** | | | | | |
| p16 | cyclin-dependent kinase inhibitor 2A | 1.15  (0.1487) | 1.66 | 0.81  (0.0123) | -2.38 |
| p21 | cyclin-dependent kinase inhibitor 1A | 1.12  (0.1543) | 1.10 | 0.52  (<0.0001) | -5.13 |
| TP53 | tumor protein p53 | 0.96  (0.1522) | -0.75 | 0.86  (0.0165) | -1.98 |
| **SASP GROWTH FACTORS** | | | | | |
| IGFBP2 | insulin like growth factor binding protein 2 | 1.31  (0.0882) | 2.41 | -0.82  (0.0003) | -1.86 |
| IGFBP4 | insulin like growth factor binding protein 4 | 1.28  (0.0633) | 2.61 | -0.65  (0.0153) | -4.97 |
| IGFBP5 | insulin like growth factor binding protein 5 | **1.78**  **(<0.0001)*** | 4.70 | **2.02**  **(<0.0001)*** | 7.61 |
| PAPPA | pregnancy-associated plasma protein A, pappalysin 1 | 0.98  (0.0102) | -0.12 | 1.78  (<0.0001)* | 4.14 |
| PDGFA | platelet-derived growth factor alpha polypeptide | 0.88  (0.1231) | -1.43 | 0.32  (<0.0001) | -12.31 |
| PDGFB | platelet-derived growth factor beta polypeptide | 0.78  (0.0577) | -2.40 | 0.45  (<0.0001) | -9.47 |
| TGFB2 | transforming growth factor beta 2 | 1.04  (0.1604) | 0.29 | 0.82  (0.0003) | -1.54 |
| TGFB3 | transforming growth factor beta 3 | 1.24  (0.1368) | 2.01 | 1.57  (<0.0001)* | 4.14 |
| VEGF-A | vascular endothelial growth factor A | 0.88  (0.1360) | -1.12 | 0.35  (<0.0001) | -11.21 |
| **SASP MATRIX REMODELLING** | | | | | |
| ACTA2 | actin, alpha 2, smooth muscle, aorta | **1.51**  **(<0.0001)*** | 3.87 | **1.55**  **(<0.0001)*** | 4.06 |
| COL1A1 | collagen, type I, alpha 1 | 1.35  (0.1429) | 1.70 | 5.92  (<0.0001)* | 14.45 |
| MMP2  (Pro-fibrotic) | matrix metallopeptidase 2 | 1.33  (0.0474)* | 2.79 | 1.01  (0.0010) | 0.14 |
| MMP3  (Pro-fibrotic) | matrix metallopeptidase 3 | 1.04  (0.1604) | 0.26 | 1.31  (0.0003)* | 2.29 |
| MMP7  (Pro-fibrotic) | matrix metallopeptidase 7 | 2.23  (0.0843) | 2.44 | 5.22  (<0.0001)* | 6.05 |
| MMP9  (Pro-fibrotic) | matrix metallopeptidase 9 | 1.21  (0.1604) | 0.84 | 0.97  (0.0003) | -0.13 |
| MMP10 | matrix metallopeptidase 10 | 1.48  (0.1512) | 1.55 | 2.42  (<0.0001)* | 4.15 |
| MMP12  (Pro-fibrotic) | matrix metallopeptidase 12 | 1.43  (0.1522) | 1.40 | 2.16  (<0.0001)* | 3.50 |
| MMP13  (Anti-fibrotic) | matrix metallopeptidase 13 | 2.05  (0.0706) | 2.58 | 3.94  (<0.0001)* | 6.12 |
| PN | periostin (osteoblast specific factor) | 1.51  (0.1301) | 2.10 | 10.51  (<0.0001)* | 12.87 |
| OPN | osteopontin (secreted phosphoprotein 1) | 2.90  (0.0633) | 2.60 | 15.57  (<0.0001)* | 9.45 |
| VCAM1 | vascular cell adhesion molecule 1 | 0.74  (0.1197) | -1.49 | 2.36  (<0.0001)* | 4.83 |

SASP = senescence-associated secretory phenotype. d = standardized change in expression (relative difference

**Table S6**. Summary of immunohistochemistry findings in IPF and NSIP.

| **Antibody** | **Cellular Localization** | **Expression** | **IPF/NSIP** |
| --- | --- | --- | --- |
| **p16** | Nuclear and cytoplasmic | Metaplastic bronchial epithelium and in fibroblastic foci | Positive fibroblastic foci in 68% of IPF.  No positive fibroblastic foci in NSIP cases |
| **p53** | Nuclear | Epithelium and interstitial fibroblasts | Expressed in both groups |
| **Periostin** | Extracellular | Areas of fibrosis | Expressed in both groups |
| **Osteopontin** | Extracellular | Lumens of remodeled air spaces | Increased expression in cases of IPF |

**Table S7**. Receiver operating characteristic (ROC) analysis of serum biomarkers vs. 12-month clinical progression.

| **Biomarker** | **Are under**  **the curve** | **Sensitivity**  (95% C.I.) | **Specificity**  (95% C.I.) | **p value** |
| --- | --- | --- | --- | --- |
| **Periostin** (ng/ml)  **Periostin** (>338 ng/ml or increase >338 ng/ml from baseline) | 0.73  0.76 | 68  (43-87)  79  (54-94) | 80  (52-96)  73  (45-92) | 0.0107  0.0025 |
| **Osteopontin** (ng/ml) | 0.61 | 70  (35-93) | 56  (21-86) | n.s. |
| **KL-6** (ng/ml) | 0.50 | 89  (67-99) | 33  (12-62) | n.s. |
| **MMP-9** (ng/ml) | 0.63 | 32  (13-57) | 100  (77-100) | n.s. |
| **Surfactant protein A** (pg/ml) | 0.60 | 100  (81-100) | 29  (8-58) | n.s. |
| **CCL-18** (ng/ml) | 0.55 | 58  (33-80) | 67  (38-88) | n.s. |

**SUPPLEMENTAL FIGURE LEGENDS**

**Figure S1**. A. P**robe intensity histogram.** B. F ratio: signal-to-noise ratio, IPF vs. NSIP analysis across the whole genome. The bar indicates the average signal for all genes. The height of the bar is the mean square. UIP=IPF.

**Figure S2**. Principal component analysis, a global analysis across the whole genome. IPF and NSIP groups are shown. UIP=IPF.

**Figure S3**. Comparison of gene expression levels determined by oligonucleotide microarray (left) and by quantitative RT-PCR (right; ratio with the expression the housekeeping gene GADPH) in the IPF and NSIP groups: A. MMP-7. B: OPN. C. IGFBP-5.

**Figure S4**. Ingenuity network analysis, networks with highest scores. Networks were scored based on the number of network eligible molecules they contained. Network eligible molecules with relatively increased expression are shown in red, whereas molecules with relatively reduced expression are shown in green. The intensity of the color is proportional to the fold change. Non-colored nodes represent genes added by Ingenuity pathway analysis based on its network algorithm but not upregulated in the actual microarray data. A. Network “Connective tissue disease, organismal injury and abnormality, cancer” (score=29). Network eligible molecules with relatively increased expression in the IPF group are shown in red, whereas molecules with relatively increased expression in the NSIP group are shown in green. B. Network “Anti-microbial response, inflammatory response and cancer” (score=42). Network eligible molecules with relatively increased expression I the NSIP group are shown in red.
